# A Co^II^-Hydroxide Complex That Converts Directly to a Co^II^-Acetamide during Catalytic Nitrile Hydration

**DOI:** 10.1021/acs.inorgchem.4c00754

**Published:** 2024-04-12

**Authors:** Philipp Heim, Sachidulal Biswas, Hugo Lopez, Robert Gericke, Brendan Twamley, Aidan R. McDonald

**Affiliations:** † School of Chemistry, Trinity College Dublin, The University of Dublin, College Green, Dublin 2, Ireland; ‡ Helmholtz-Zentrum Dresden-Rossendorf e.V., Institute of Resource Ecology, Bautzner Landstraße 400, 01328 Dresden, Germany

## Abstract

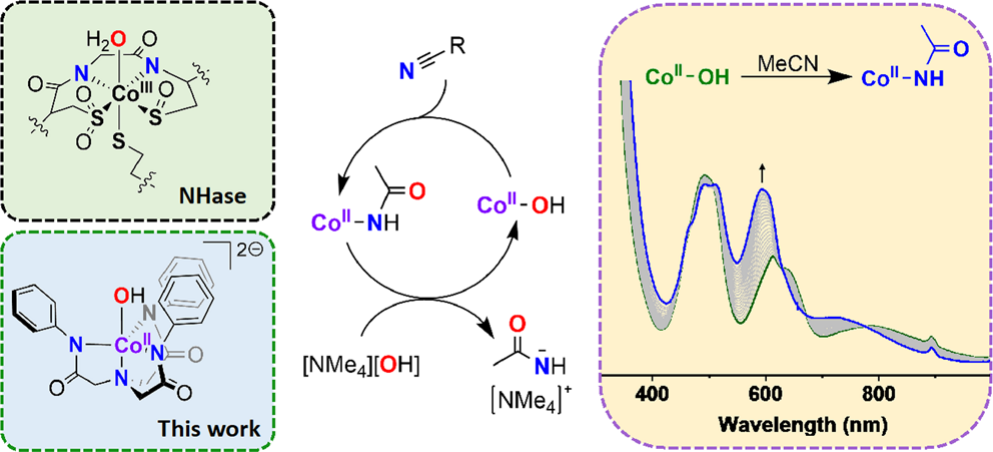

In exploring structural and functional mimics of nitrile hydratases, we report the synthesis of the *pseudo*-trigonal bipyramidal Co^II^ complexes (K)[Co^II^(DMF)(L^Ph^)] (**1(DMF)**), (NMe_4_)_2_[Co^II^(OAc)(L^Ph^)] (**1(OAc)**), and (NMe_4_)_2_[Co^II^(OH)(L^Ph^)] (**1(OH)**) (L^Ph^ = 2,2′,2’’-nitrilo-*tris*-(*N*-phenylacetamide; DMF = *N*,*N*-dimethylformamide; ^–^OAc = acetate)). The complexes were characterized using NMR, FT-IR, ESI-MS, electronic absorption spectroscopy, and X-ray crystallography, showing the L^Ph^ ligand to bind in a tetradentate tripodal fashion alongside the respective ancillary donor. One of the complexes, **1(OH)**, is an unusual structural and functional mimic of the Co active site in Co nitrile hydratases. **1(OH)** reacted with acetonitrile to yield the Co^II^-acetamide complex (NMe_4_)_2_[Co^II^(NHC(O)CH_3_)(L^Ph^)], **2**, which was also thoroughly characterized. In the presence of excess hydroxide, **1(OH)** was found to catalyze quantitative conversion of the added hydroxide into acetamide. Despite the differences in Co oxidation state in nitrile hydratases and **1(OH)** (Co^III^ versus Co^II^, respectively), **1(OH)** was nonetheless an effective nitrile hydration catalyst, selectively producing acetamide over multiple turnovers.

## Abstract 2

A Co^II^-hydroxide complex reacted with acetonitrile to yield the corresponding Co^II^-acetamide complex. In the presence of excess hydroxide, the reaction with acetonitrile was catalytic with the quantitative conversion of the added hydroxide into acetamide. Despite the difference in Co oxidation state in Co NHase (Co^III^), the Co^II^ synthetic model was nonetheless an effective nitrile hydration catalyst.

## Introduction

Nitrile hydratases (NHase) catalyze the hydration of organic nitriles to yield the corresponding amides.^1−3^ Nitriles are generally difficult to hydrate in the absence of a catalyst and the hydration reaction is troublesome to control, conversion to carboxylic acid rather than amide being often observed. NHase, with their ability to selectively convert nitrile to acetamide, have found industrial applications for the preparation of nicotinamide (among others), one of very few examples of an industrial application of metalloenzymes.^4,5^ The active site of NHase contains either a mononuclear Fe or Co ion coordinated by two carboxamidate N-donors and multiple cysteinate or oxygenated cysteine (sulfinate or sulfenate) ligands.^3^ Interestingly, the oxidation state assigned to the Fe and Co ions in NHase was always +3, suggesting that nature has evolved to employ a relatively electron-poor metal catalyst to mediate nitrile activation.

Synthetic models that mimic both the structure and function of Co^III^ NHase have been reported,^3,6−10^ although they tend to be poor catalysts, demonstrating limited efficacy in multiturnover nitrile hydration.^11,12^ Employing mononuclear Co^II^ complexes as catalysts is very rare, with limited insight into the role of the Co^II^ ion, the metal-bound ligands, the metal-bound products, and the mechanism of nitrile activation.^13−16^ For example, the direct conversion of a mononuclear Co^II^-hydroxide adduct to Co^II^-acetmide has not been reported, to the best of our understanding. The examples of mononuclear Co^II^ catalysts capable of nitrile hydration provide limited mechanistic insight, nor do they demonstrate the role of metal-bound ligands.

Two general mechanistic postulates defining the role of the metal ion in NHase have been made:^6^ (a) the metal would act as a Lewis acid to activate a metal-bound nitrile to attack by hydroxide; (b) the metal would act to activate a metal-bound hydroxide ligand for nucleophilic attack on a nitrile group. Further nuance is likely: both hydroxide and nitrile could be metal-bound. Unfortunately, a consensus has not been reached, with synthetic models containing metal-bound nitrile and hydroxide ligands both demonstrating the ability to hydrate nitriles. Consideration of the oxidation state of the Co ion would favor mechanism (a), where a more Lewis acidic Co^III^ may render a metal-bound nitrile highly active. In contrast, a Co^II^ ion would be considered more Lewis basic and thus may favor nucleophilic attack by a metal-bound hydroxide ligand (thus mechanism (b)). Herein, we report the preparation of a Co^II^-hydroxide complex supported by a polycarboxamidate ligand that was capable of on-complex catalytic nitrile-to-acetamide conversion. The Co^II^–OH complex was not only a reasonable structural and functional mimic of NHase but also an effective catalyst.

## Results and Discussion

**Scheme 1:**
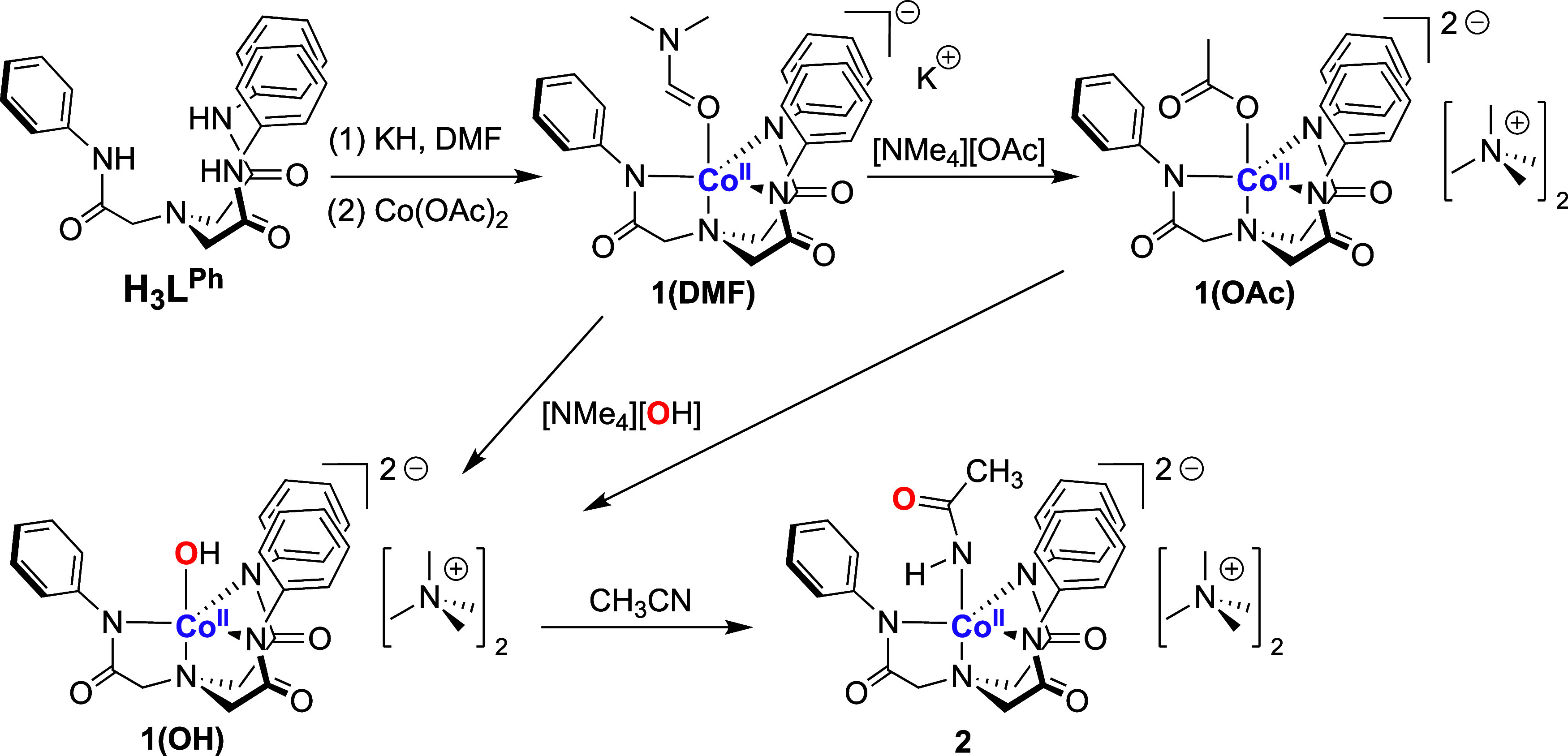
Preparation of **1(DMF)**, **1(OAc)**, **1(OH)**, and **2**

Addition of KH to 2,2′,2″-nitrilo-*tris*-(*N*-phenylacetamide) (L^Ph^) in *N*,*N*-dimethylformamide (DMF) resulted in the evolution of a gas (Scheme 1, see the Supporting Information for details). Treatment of the resultant (presumed but not isolated) K_3_[L^Ph^] salt with anhydrous Co^II^(OAc)_2_ (OAc = acetate) resulted in an immediate color change from pale yellow to dark purple. The purple species was identified as K[Co^II^(DMF)(L^Ph^)] (**1(DMF)**), obtained in 42% yield. Addition of tetramethylammonium acetate ([NMe_4_][OAc]) to a DMF solution of **1(DMF)** yielded [NMe_4_]_2_[Co^II^(OAc)(L^Ph^)] (**1(OAc)**) as a purple product in 61% yield. Alternatively, tetramethylammonium hydroxide ([NMe_4_][OH]) in CH_3_OH was added to **1(DMF)** in DMF, causing an immediate color change from dark purple to dark green. After workup, a green complex, [NMe_4_]_2_[Co^II^(OH)(L^Ph^)] (**1(OH)**) was obtained in 85% yield.

**Figure 1:**
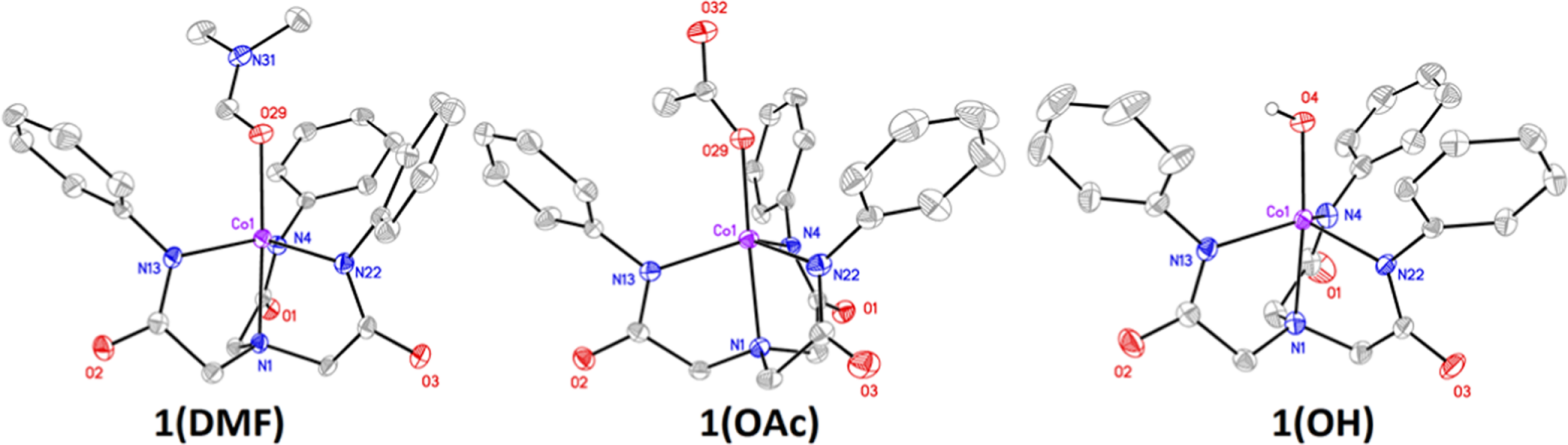
ORTEPs of **1(DMF)**, **1(OAc)**, and **1(OH)**. Plotted at the 50% probability level. Hydrogen atoms, with the exception of the hydroxide in **1(OH)**, solvent molecules, and counterions have been omitted for clarity. The hydroxide hydrogen atom in **1(OH)** was placed in an idealized position.

**1(DMF)**, **1(OAc)**, and **1(OH)** were crystallized via layering of diethyl ether (Et_2_O) into concentrated CH_3_CN or DMF solutions of the complexes and allowing for slow crystallization at room temperature (see the Supporting Information for details). Single-crystal X-ray diffraction analysis of **1(DMF)** revealed a *pseudo*-trigonal bipyramidal (TBP) coordination geometry (Figure 1 and Table S1 and Figures S1–S4), with L^Ph^ acting as a tripodal trianionic tetradentate ligand and a single DMF molecule coordinated via an O-atom in the fifth ligand site (Co–O = 2.11(7) Å, Table 1). K^+^ counterions interacted with the amidate carbonyl groups of the L^Ph^ backbone, bridging four units of **1(DMF)** in the unit cell. Structural analysis of **1(OAc)** revealed the same coordination of L^Ph^ with the Co^II^ ion as in **1(DMF)**. We noted the displacement of the ancillary DMF ligand with an ^–^OAc ligand and the presence of two ^+^[NMe_4_] cations for each [Co^II^(OAc)(L^Ph^)]^2–^ unit. A monodentate binding mode for ^–^OAc was observed (Co–O = 2.03(1) Å) with the second O-atom too far away to indicate any interaction with the Co ion. The monodentate binding mode was previously observed in the analogous [Fe^II^(OAc)(L^Ph^)]^2–^ and [Zn^II^(OAc)(L^Ph^)]^2–^ complexes reported by Lacy et al.;^17^ however, this contrasts to the bidentate mode observed in [Ni^II^(OAc)(L^Ph^)]^2–^.^18^ An apical coordination of a hydroxide O-atom was observed in the structure of **1(OH)** (Co–O = 1.94(7) Å), falling within the range of reported Co–OH complexes with similar coordination modes.^16,19−24^ A CCDC search of Co–OH complexes containing four N-donors resulted in 14 hits, where the average Co–O distance was 1.97(8) Å (Figure S5).^25^ We concluded that **1(OH)** was a hydroxide complex and not an aqua adduct (supported by the fact that two ^+^[NMe_4_] cations were observed for each [Co^II^(OH)(L^Ph^)] unit). The average Co–O distance in Co–OH_2_ complexes were also significantly longer (2.13(8) Å, based on a data set of 17 compounds).^24,26−33^ For the set of three complexes, a narrow range for the geometry index was noted (τ_5_ = 0.76–0.82), where 1.0 presents a perfect TBP geometry and 0.0 represents square based pyramidal geometry.^34^ The discrepancies are indicative of the minor steric influences of the different ancillary (5th) ligands, leading to slight distortions in the overall structures. Finally, the more basic ^–^OH ligand yielded a shorter Co–O bond (1.94 Å, Table 1) than the less basic DMF and ^–^OAc ligands (2.11 and 2.03 Å, respectively), indicating a stronger Co/O interaction in moving through from weak field DMF, stronger field ^–^OAc, to strongest field ^–^OH ligands. Finally, the *tris*-amidate equatorial coordination provided by L^Ph^ provides a reasonably good structural mimic for the *bis*-amidate *mono*-cysteinate equatorial binding to Co in the NHase active site.^3^

**Table 1:** Spectral, Magnetic, and Structural Properties of **1(DMF)**, **1(OAc)**, **1(OH)**, and **2**

	λ_max,_ nm (ε, mol L^–1^ cm^–1^)	μ_eff_ (Bohr magneton)	*E*_pa_ (V)^c^	Co–O/N (Å)^d^	τ_5_
**1(DMF)**^a^	529 (320)	3.35	–0.26	2.11(7)	0.82(5)
**1(OAc)**^b^	473 (120), 500 (140)	3.26	–0.32	2.03(1)	0.80(8)
**1(OH)**^b^	500 (150), 650 (70), 790 (25)	3.99	–0.21	1.94(7)	0.76(5)
**2**^b^	500 (160), 593 (90)	3.43	–0.22	2.02(3)	0.80(5)

a DMF was solvent.

b CH_3_CN was solvent.

c *E*_pa_ = peak anodic potential, used because the CV for **1(OAc)** appeared irreversible. Calibrated against the ferrocene/ferrocenium (Fc/Fc^+^) couple measured under the same conditions.

d As determined by X-ray diffraction measurements. Corresponding to O/N atom of ancillary 5th ligand, not L^Ph^.

ESI-MS spectra measured on solutions of **1(DMF)**, **1(OAc)**, and **1(OH)** displayed the most prominent peak at *m*/*z* = 472.09 ([Co^II^(L^Ph^)]^−^, expected *m*/*z* = 472.0946), confirming the presence of the mono anionic [Co^II^(L^Ph^)]^−^ core in each complex. A low-intensity peak found at *m*/*z* = 531.1072, (assigned to [Co(OAc)(L^Ph^)]^−^, expected *m*/*z* = 531.1079) was observed for **1(OAc)** (Figure S6). With the exception of **1(OAc)**, the ancillary fifth ligand could not be identified in ESI-MS studies, presumably due to the hard ionization technique, which caused fragmentation of the complexes. Overall, ESI-MS confirmed that the [Co(L^Ph^)] entity was stable in the solution state.

**Figure 2:**
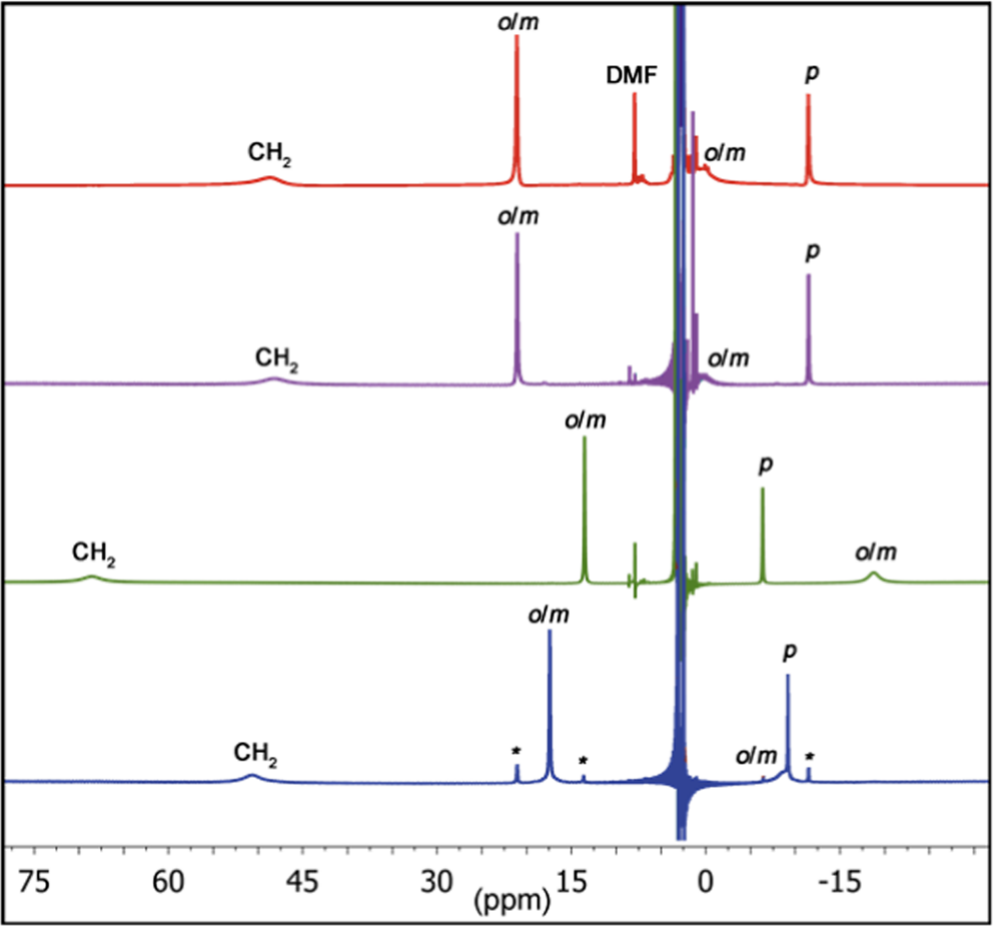
^1^H NMR spectra of **1(DMF)** (red trace), **1(OAc)** (purple), **1(OH)** (green), and **2** (blue). All measurements were in [D_6_]-DMSO at 20 °C.

^1^H nuclear magnetic resonance (NMR) spectra of **1(DMF)**, **1(OAc)**, and **1(OH)** displayed four broadly shifted peaks in the range of −20 to +70 ppm for each complex (Figures 2 and S7–S9). Integration of the peaks gave an approximate 2:2:2:1 ratio corresponding to the H atoms in L^Ph^ assigned to the CH_2_, and *ortho-*, *meta-*, and *para*-CH arene positions, respectively. Sharp upfield resonances at δ = ca. −5 to −15 ppm were assigned to the *para-*CH of the phenyl ring based on their integration value. Broader signals at δ ∼ 0 to 25 ppm and sharper resonances at +10 to 20 ppm were assigned to either the *ortho-*CH or *meta-*CH positions. The most downfield shifted signals at δ ∼45 to 70 ppm were assigned to the CH_2_ signal of the methylene bridge on L^Ph^. This assignment was made based on the assignments of CH signals in [Ni^II^(L^Ph^)(OAc)]^2–^ and its deuterated analogue [Ni^II^(D_15_-L^Ph^)(OAc)]^2–^ (D_15_-L^Ph^ = 2,2′,2″-nitrilo-tris(*N*-2,3,4,5,6-[*D*]-phenyl)acetamide) where a per-deuterated form of L^Ph^ was used to identify the resonances.^18^ All other peaks were assigned to free solvent (DMF, δ = 2.72, 2.90, and 7.97 ppm). The appearance of these signals in the diamagnetic (0–12 ppm) range and at the natural frequency of the free solvent suggests that some free DMF was in solution in **1(DMF)**. For **1(OAc)**, we observed no resonance that could be assigned to the CH_3_ of a metal-bound ^–^OAc group. We have made similar observations for [Ni^II^(OAc)(L^Ph^)]^2–^,^18^ as have others for [Fe^II^(OAc)(L^Ph^)]^2–^,^17^ where the ^–^OAc group could not be identified by ^1^H NMR. Free ^–^OAc could be identified at δ = 1.44 ppm, suggesting that some free ^–^OAc was present. Interestingly, the electronic absorption spectra of **1(DMF)** and **1(OAc)** in DMSO were not the same (Figure S10). Thus, despite the similarities in their ^1^H NMR spectra, the ancillary anionic fifth ligand has not been displaced in these complexes. For **1(OH)**, a peak for the OH signal could not be identified; however, the CH resonances displayed significant shifts compared to those obtained for **1(DMF)** and **1(OAc)** suggesting that the OH ligand was Co-bound in **1(OH)** in solution.

A solution state magnetic moment was determined via the Evans method at 20 °C.^35^ The three complexes displayed effective magnetic moments in the range of μ_eff_ = 3.3–4.0 (Table 1), corresponding to complexes with three unpaired electrons associated with the Co^II^ ion. Assuming a *pseudo*-TBP geometry was maintained in solution, this value is consistent with a high spin *S* = 3/2 configuration of a d^7^ Co^II^ ion. The unpaired electrons are expected to be located in metal-type d_z2_ and degenerate d_x2-y2_/d_xy_ orbitals. **1(OH)** displayed an electronic absorption feature at λ = 790 nm. Such near-IR absorption features are known for Co^II^–OH complexes (λ = 700–900 nm), and have been assigned to d-d transitions typically displaying extinction coefficients (ε) of <50 mol L^–1^ cm^–1^.^19,23^ This feature has been predicted to stem from a ^4^A_1_ → ^4^E transition in *S* = 3/2 Co^II^ complexes in *C*_3*v*_ symmetry (as identified here for **1(OH)**). Interestingly, for the weaker-field axial fifth donors in **1(DMF)** and **1(OAc)**, this band appears to be blue-shifted (Figure S10), consistent with observations made previously where the ligand field was found to influence this transition.^19,23^ Taken together, the data suggest that the solution structure is in agreement with the solid-state structures.

Fourier transform infrared (FT-IR) spectra of **1(DMF)**, **1(OAc)**, and **1(OH)** showed the loss of ν_N–H_ of the amide in L^Ph^ (ν = 3237 cm^–1^, Figure S11),^17^ indicating a deprotonation and complexation of the ligand had occurred. **1(DMF)** displayed a peak at ν = 1663 cm^–1^, tentatively assigned to ν_C=O_ of bound DMF. We were tentative in the assignment because L^Ph^ contains three C=O groups that potentially display vibrational modes in the same window. **1(OAc)** showed features at ν = 1560 cm^–1^ and ν = 1375 cm^–1^ (Δν = 185 cm) that were (again tentatively) assigned to ν_a/as_ modes of bound ^–^OAc. The observed difference in ν_a/as_ was larger than that observed in free ^–^OAc (Δν = 163 cm^–1^), suggesting a monodentate coordination mode of the ^–^OAc anion, in agreement with our XRD analysis.^36^ For **1(OH)**, a peak at ν = 3503 cm^–1^ was assigned to the ν_O–H_ and was consistent with what is typically observed for Co–OH complexes.^37^ FT-IR thus confirmed the XRD and NMR assignments of **1(DMF)**, **1(OAc)**, and **1(OH)**.

Steady-state cyclic voltammetry of **1(DMF)**, **1(OAc)**, and **1(OH)** was performed in DMF at room temperature using [^n^Bu_4_N][PF_6_] as supporting electrolyte (Figure S12 and Table 1). With the exception of **1(OAc)**, the complexes displayed either fully or quasi-reversible redox events at ca. −0.3 V versus the ferrocene/ferrocenium couple. We have assigned these peaks to the Co^II^/Co^III^ redox couple, based on the range of peak anodic potentials measured between −0.32 and −0.21 V in DMF, for a series of similar tripodal Co^II^ complexes supported by tris-anionic donor ligands.^37^ Little difference in the Co^II^/Co^III^ redox couple was observed despite exchanging neutral DMF for more basic (anionic) ^–^OAc and ^–^OH donors. Mn and Fe complexes supported by L^Ph^ have displayed similarly unchanged potentials, despite changes to the electron-donating properties of the supporting ligand.^38^ The highest *E*_ox_ was observed for **1(OH)** suggesting the most electron deficient Co^II^ center, while **1(OAc)** would appear to contain the most electron-rich Co^II^ site. However, the differences among the sets of three are minimal, suggesting little difference in their redox properties.

### Reaction of **1(OH)** with CH_3_CN

**Figure 3:**
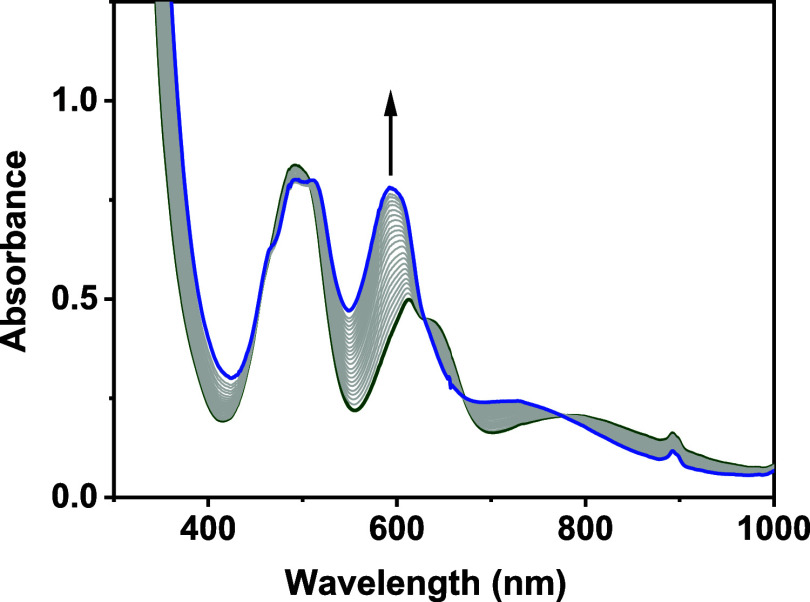
Conversion of **1(OH)** (green trace, 5.0 mM in CH_3_CN) to **2** (blue trace) which formed over the course of 5 h at 20 °C. Gray traces show incremental changes in the electronic absorption properties.

We were interested in exploring the efficacy of **1(OH)** as a mimic for Co NHase. We monitored the reaction of **1(OH)** with CH_3_CN by electronic absorption spectroscopy (Figures 3 and S13). Stirring of a freshly prepared CH_3_CN solution of **1(OH)** resulted in the blue-shifting of the near-IR band assigned to **1(OH)** and a concomitant increase of a new absorption feature at λ = 593 nm assigned to a new species, defined as **2**. The loss of the near-IR band typical of Co^II^–OH complexes and reversion to an electronic absorption spectrum similar to **1(OAc)**, suggested that the new species contained a weaker-field ancillary donor ligand. The reaction was complete within 5 h (Figure S14). We observed isosbestic points at λ = 630, 670, and 775 nm, consistent with a clean conversion of **1(OH)** to **2**. This indicated that the formation of **2** resulted from a reaction between **1(OH)** and CH_3_CN, leading us to conclude that indeed **1(OH)** was reacting with the solvent (CH_3_CN) to yield a presumed hydrated product.

**Figure 4:**
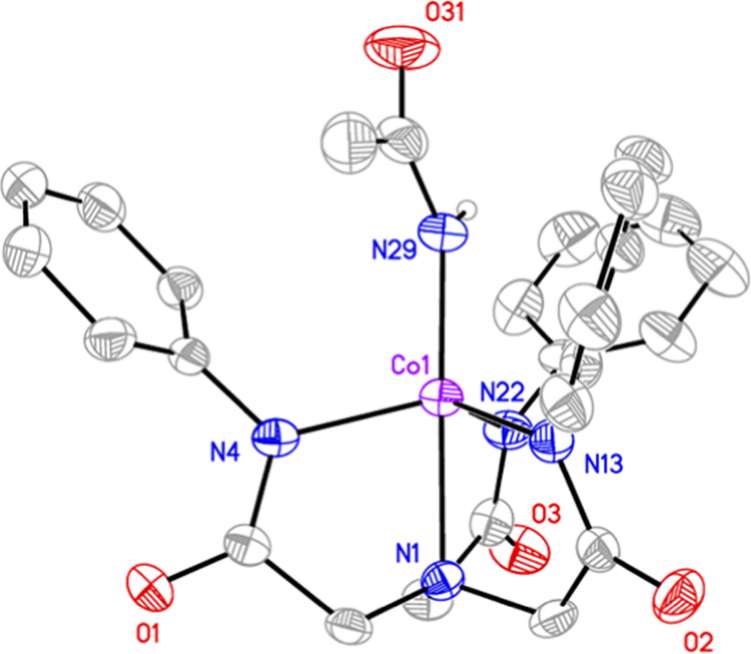
ORTEP of compound **2**. Plotted at 50% probability level. Hydrogen atoms (apart from the acetamide NH), solvent molecules, and counterions have been omitted for clarity. The acetamide hydrogen atom was placed in an idealized position.

Notably, when we attempted to crystallize the green complex **1(OH)** in CH_3_CN we observed the formation of a purple crystalline material over the course of 1 day (see the Supporting Information for details). This product was identified as acetamide complex **2** and was obtained as a dark purple solid in 41% yield (Scheme 1 and Figure 4). XRD analysis on **2** revealed a five-coordinate structure with a striking resemblance to that of the earlier reported family of complexes. The axial fifth ligand was identified as an N-bound acetamide (^−^N(H)C(O)CH_3_), leading us to define **2** as (NMe_4_)_2_[Co^II^(N(H)C(O)CH_3_)(L^Ph^)]^2–^.

The ^1^H NMR spectrum of **2** appeared different from **1(OH)**, confirming a reaction had occurred (Figures 2 and S15). Interestingly, the ^1^H NMR spectrum of **2** was more similar to those measured for **1(DMF)** and **1(OAc)**, consistent with the three complexes displaying similar structures. New peaks that could be attributed to the NH and CH_3_ resonances of the newly formed, Co-bound acetamide ligand, were not identified, consistent with previous observations for **1(DMF)**, **1(OAc)**, and **1(OH)**, where ancillary ligand’s OH and CH_3_ resonances could not be identified. Importantly, there are shifts in the resonances associated with **2** when compared to **1(DMF) 1(OAc)**, and **1(OH)**, suggesting that the acetamide ligand induces small changes in the ^1^H NMR data, consistent with it being bound in solution. FT-IR analysis of **2** showed the absence of the ν_O–H_ = 3503 cm^–1^ peak attributed to the O–H stretch in **1(OH)**, and the formation of a higher-energy feature at ν = 3238 cm^–1^ for **2** (Figure S11). We assigned this new feature to the ν_N–H_ of the newly formed acetamide group, which was consistent with a previous report on Co-acetamide complexes (ν_N–H_ ∼ 3410 cm^–1^).^16^ Postreaction ESI-MS analysis from the reaction of **1(OH)** with CH_3_CN showed the formation of acetamide (Figure S16), confirming the release of free acetamide from the reaction. Thus, although a molecular ion peak for **2** was not observed, there is evidence of the formation of acetamide through ESI-MS. In summary, our NMR, FT-IR, and ESI-MS analyses support the assignment (from XRD measurements) of **2** as a Co^II^-acetamide complex derived from acetonitrile.

To explore the catalytic efficacy of the hydroxide complex **1(OH)** in nitrile hydration, we reacted **1(OH)** with a large excess of [NMe_4_][OH] (20 equiv) in CH_3_CN. The solution was then stirred under an inert atmosphere for 48 h at room temperature (20 °C). Following an aqueous workup, free acetamide was detected at the end of the reaction by ^1^H NMR analysis (Figure S17). The yield of acetamide was found to be quantitative (thus a turnover number, TON = 20; after 24 h, the TON = 13). Furthermore, when 100 equiv. [NMe_4_][OH] was added to a CH_3_CN solution of **1(OH)**, we observed quantitative conversion to acetamide within 65 h (TON = 100, Figure S18). The hydration reaction was thus slow but always quantitative. Importantly, we found that under aerobic conditions, the catalyst was less stable and stopped turning over after 6 h. Under anaerobic conditions, **2** was always the product of the catalyzed reaction, whereas **2** was observed to decay under aerobic conditions and stopped turning over. Our general observation was that as long as air was excluded from the reaction mixture, all [NMe_4_][OH] that was added was turned over into acetamide. Finally, we found that **1(OH)** also reacted with benzonitrile and butyronitrile to yield products that displayed spectral properties similar to those of **2** (thus both substrates were hydrated to the corresponding acetamide, Figure S19).

**1(OH)** was a reasonable structural mimic for the Co active site in NHase in that it provided a *tris*-anionic first coordination sphere (three amidates in **1(OH)** versus two amidate and a cysteinate in NHase). Unlike Co NHase, which contains a Co^III^ ion, **1(OH)** contained a Co^II^ ion. The Co ion in **1(OH)** was thus likely to be less Lewis acidic. Despite this, **1(OH)** was a reasonable functional mimic of Co NHase, efficiently converting nitriles into metal-bound acetamides. We postulate that the metal-bound hydroxide ligand is rendered highly nucleophilic by the Co^II^ ion *and* the *tris*-anionic first coordination sphere. Given that isosbestic points were observed in the conversion of **1(OH)** to **2**, we tentatively assume that the nitrile group that is attacked was bound, although it is equally plausible that nitrile was not coordinated to the Co ion. We note there is a vacant “6th” site on the Co^II^ ion, where nitrile could bind. The current example demonstrates that from a mechanistic perspective, rendering metal-bound hydroxide ligands highly nucleophilic through a strongly donating trianionic supporting ligand *and* lower metal oxidation state will generate an effective nitrile hydration catalyst. Overall, **1(OH)** represents an unexpected example of a mononuclear Co NHase structural and functional mimic, where, despite the lower Co oxidation state, effective nitrile hydration to acetamide was achieved with multiple turnovers.

## Conclusions

A series of mononuclear Co^II^ complexes, **1(DMF)**, **1(OAc)**, and **1(OH)**, were synthesized and characterized using X-ray diffraction, NMR, FT-IR, and electronic absorption spectroscopies. The complexes were supported by the *tris*-carboxamidate ligand L^Ph^, which bonded in a tripodal fashion yielding Co^II^ ions in a *pseudo*-trigonal bipyramidal coordination environment. One of the complexes, **1(OH)**, was found to be a reasonable structural and functional mimic of the Co active site in Co NHase. **1(OH)** reacted with acetonitrile to yield Co^II^-acetamide complex **2**, which was also thoroughly characterized. In the presence of excess hydroxide, **1(OH)** was found to catalyze the quantitative conversion of added hydroxide with acetonitrile into acetamide. Despite the differences in Co oxidation state in NHase and **1(OH)** (Co^III^ versus Co^II^, respectively), **1(OH)** was nonetheless an effective nitrile hydration catalyst, selectively producing multiple turnovers of acetamide in acetonitrile hydration.

## Supporting Information Available

Safety statement; experimental and physical methods; NMR, electronic absorption, and IR spectra; cyclic voltammetry data; mass spectrometry data; crystallographic information; and kinetics data and analysis (PDF)
