# Supplementary material for: A CoII-Hydroxide Complex That Converts Directly to a CoII-Acetamide during Catalytic Nitrile Hydration
Source: Inorg Chem. 2024 Apr 12;63(17):7896–902. doi: 10.1021/acs.inorgchem.4c00754 (PMC11061833; doi:10.1021/acs.inorgchem.4c00754)
Supplement: Supplementary file 1 — ic4c00754_si_001.pdf [file ic4c00754_si_001.pdf]

Supporting Information for:

A Co^{II}-hydroxide complex that converts directly to a Co^{II}-acetamide during catalytic nitrile hydration

Philipp Heim,[1] Sachidulal Biswas,[1] Hugo Lopez,[1] Robert Gericke,[1,2] Brendan Twamley,[1] Aidan R. McDonald*[1]

[1]School of Chemistry, Trinity College Dublin, the University of Dublin, College Green, Dublin 2, Ireland

[2] Current address: Helmholtz-Zentrum Dresden-Rossendorf e.V., Institute of Resource Ecology, Bautzner Landstraße 400, 01328 Dresden, Germany

Email: aidan.mcdonald@tcd.ie

Physical methods:

NMR analysis was performed on an Agilent MR 400 MHz NMR spectrometer. The solution state *Evans method* was used to probe the number of unpaired electrons associated with each complex.[1] Electrospray ionization (ESI) mass spectra were acquired using a Micromass time of flight spectrometer (TOF), interfaced to a Waters 2690 HPLC, or by direct injection in the mass spectrometry instrument. Electronic absorption spectra were recorded using an Agilent 8453 diode array spectrophotometer (190 – 1100 nm range), attached to a Unisoku Scientific Instrument cryostat using liquid nitrogen as coolant (where needed). Attenuated total reflectance Fourier transform infra-red (ATR-FTIR) spectra were recorded on a Perkin-Elmer Spectrum 100 Fourier transform infrared spectrometer.

Cyclic voltammetry: Steady state cyclic voltammetry experiments were conducted on 1.0 mM solutions of complex in DMF at room temperature, using glassy carbon as a working electrode, an $Ag/AgNO_3$ reference electrode, and Pt wire as the counter electrode. $[^nBu_4N][PF_6]$ (0.1 M) was used as the supporting electrolyte. A 0.05 V s^{-1} scan rate was used throughout. All data were referenced against the ferrocene/ferrocenium (Fc/Fc^+) measured under the same conditions.

Single crystal X-Ray diffraction: Data for **1(DMF)**, **1(OAc)**, were measured on a Bruker D8 Quest ECO using Mo Kα radiation (λ = 0.71073 Å) and data for **1(OH)** and **2** were collected on a Bruker APEX DUO using Mo and Cu Kα radiation (λ = 1.54178 Å). Each sample was mounted on a MiTeGen cryoloop and data collected at 105(2) K for **1(DMF),** 100(2) K for **1(OAc)**, **1(OH)** and 150(2) K for **2** using and Oxford Cryosystems Cryostream or Cobra low temperature device. Bruker APEX[2] software was used to collect and reduce data and correct

for Lorentz and polarization effects. Absorption corrections were applied using SADABS.[3] Structures were solved with the XT structure solution program[4] using Intrinsic Phasing. All were refined using Least Squares method on F^2 with XL.[5] All non-hydrogen atoms were refined anisotropically. Hydrogen atoms were assigned to calculated positions using a riding model with appropriately fixed isotropic thermal parameters. Molecular graphics were generated using OLEX2[6] and the asymmetric unit with heteroatoms labelled is shown in Fig. S1-S4. Crystal data, details of data collection and refinement are given in Table S1. **1(DMF)**: Hydrogen atoms on C2, C11 and C20 were located on the difference map and refined semi-free with coordinates riding on the carrier atom (AFIX). **1(OH)**: Hydrogen atoms on C2, C11 and C20 were located on the difference map and refined semi-free with coordinates riding on the carrier atom (AFIX). The OH hydrogen was located on the difference map and refined semi-free with restraints (DFIX). An unknown solvent(s) was(were) removed using the routine SQUEEZE in PLATON.[7] This removed 30 electrons in a void space of 132 $Å^3$ in the asymmetric unit. Crystallographic data for the structures in this paper have been deposited with the Cambridge Crystallographic Data Centre as supplementary publication nos. 2305632-2305635. Copies of the data can be obtained, free of charge, on application to CCDC, 12 Union Road, Cambridge CB2 1EZ, UK, (fax: +44-(0)1223-336033 or e-mail:deposit@ccdc.cam.ac.uk).

Materials:

All reactions with air sensitive materials were carried out in an inert atmosphere glovebox or using N_2 or Ar atmosphere Schlenk line (vacuum manifold) techniques. All reagents were purchased from commercial sources and were used as received unless otherwise stated. Anhydrous *N*,*N*-dimethylformamide (DMF) was purchased and stored over 3 Å molecular sieves in an inert atmosphere. Anhydrous CH_3CN and diethyl ether (Et_2O) were dispensed from an MBRAUN Solvent Purification System (SPS-5) and deoxygenated by sparging with a flow

of N_2 through the solution. Mineral supported KH was transferred into an inert atmosphere glovebox and washed with n-hexane to remove mineral oils and was subsequently used as a dry powder. Commercially available tetramethylammonium hydroxide ([NMe_4][OH], 25% w/v CH_3OH solution) was concentrated under vacuum to remove excess CH_3OH. It was concentrated to the point where [NMe_4][OH] started to precipitate from a viscous mother liquor. The viscous mother liquor (10.4 N (titrated against HCl)) was used as prepared. 2,2',2"-nitrilo-*tris*-(N-phenylacetamide) (L^{Ph}) was prepared according to a literature procedure.[8]

Safety Statement: *Caution:* KH and KH with the mineral support removed reacts vigorously with H_2O producing H_2 gas which can ignite. All transformations with these materials were performed in an inert atmosphere glovebox (an exclusively anhydrous environment). We maintained a maximum scale of 0.2 g KH when performing the preparations described below. Standard procedures for handling flammable solvents were employed throughout this project.

Synthesis of potassium (nitrile-tris-(N-phenylacetamide)cobalt(II)(DMF)), (K)[Co^{II}(DMF)(L^{Ph})], ***1(DMF)****:*

L^{Ph} (0.2 g, 0.48 mmol) was placed in a Schlenk flask and put under vacuum for 1 h. The solid was dissolved in anhydrous DMF (4 mL) forming a colourless solution that was stirred for 30 minutes. KH (0.085 g, 2.12 mmol) was added in one portion, turning the solution yellow. Once bubbling ceased after the addition of KH, $Co(OAc)_2$ (0.085 g, 0.480 mmol) was added in one portion, yielding a dark purple mixture. The (presumed) KOAc precipitate was allowed to settle to the bottom of the flask. The supernatant was then passed through a 0.2 µm syringe filter into a clean Schlenk flask. Crystals suitable for single crystal XRD were grown *via* slow layer

diffusion of Et_2O into a solution of **1(DMF)** in DMF, yielding purple crystals after 1 day. Yield: 0.12 g (42 %).

λ_{max}/nm (DMF, ε/L mol^{-1} cm^{-1}): 529 (320).

δ_H (400 MHz, D_6-DMSO, ppm): -11.47 (s, broad, *p-Ar*), 0.40 (*s*, broad, *o/m-Ar*) 2.72 (*s*, CH_3-DMF), 2.90 (*s*, CH_3-DMF), 7.97 (*s*, CHO-DMF), 21.09 (*s*, broad, *o/m-Ar*), 48.61 (*s*, broad, C*H_2*).

ESI-MS (*m*/*z*): Found: 472.0950 ([$Co^{II}(L^{Ph})]^-$, $C_{24}H_{21}CoN_4O_3$ requires 472.0946).

ν(ATR-FTIR)/cm^{-1}: 3056, 3029, 2927, 2894, 1663, 1597, 1578, 1559, 1544, 1485, 1447, 1382, 1336, 1263, 1219, 1168, 1131, 1101, 1068, 1026, 995, 961, 912, 902, 884, 837, 757, 705, 692, 662, 556.

Synthesis of tetramethylammonium (nitrile-tris-(N-phenylacetamide)cobalt(II)acetate, $(NMe_4)_2[Co^{II}(OAc)(L^{Ph})]$, ***1(OAc)****:*

1(DMF) was generated as described above, but once the purple mixture formed, no further work-up was performed. [NMe_4][OAc] (0.32 g, 2.40 mmol, 2 equiv.) was added in one portion and the purple mixture was allowed to stir overnight. The resulting precipitate was removed through filtration through a 0.2 μm syringe filter and the product was precipitated with the addition of dry Et_2O (100 mL) to the filtered solution. The resulting mixture was decanted and the remaining purple residue was re-dissolved in dry CH_3CN (8 mL). Any remaining solid materials were filtered through a 0.2 μm syringe filter. The desired product was precipitated by adding dry Et_2O (100 mL), and the resulting purple powder was collected by filtering on a sintered glass filter. The product was further rinsed with three aliquots of dry Et_2O (10 mL)

and then dried under vacuum for 1 h. Crystals suitable for single crystal XRD were grown *via* slow layer diffusion of Et_2O into a solution of **1(OAc)** in CH_3CN. Yield: 0.49 g (yield = 61 %).

λ_{max}/nm (MeCN, ε/L mol^{-1} cm^{-1}): 473 (120), 500 (140).

δ_H (400 MHz, D_6-DMSO, ppm): -11.5 (s, broad, *p-Ar*), 0.21 (s, broad, *o/m-Ar*), 1.44 (s, AcO^-), 3.11 (s, Me_4N^+), 21.0 (*s*, broad, *o/m-Ar*), 48.2 (*s*, broad, C*H*$_2$).

ESI-MS (*m*/*z*): Found: 531.1072 ([Co(OAc)(L^{Ph})]$^-$, $C_{26}H_{24}CoN_4O_5$ expected mass: 531.1079).

ν (ATR-FTIR)/cm^{-1}: 3024 (CH), 2892 (CH), 2839 (CH), 1597, 1560 (CO), 1485, 1447, 1375 (CO), 1331, 1256, 1216, 1170, 1128, 1071, 1025, 1008, 993, 952, 903, 880, 761, 699, 654, 612, 551.

Synthesis of tetramethylammonium (nitrile-tris-(N-phenylacetamide)cobalt(II)hydroxide, *$(NMe_4)_2[Co^{II}(L^{Ph})(OH)]$,* ***1(OH)****:*

1(DMF) (1.20 mmol) was generated as described above, but once the purple mixture formed, no further work-up was performed. A solution of $[NMe_4][OH]$ (10.4 N , 0.37 mL, 3.84 mmol, 3.2 equiv.) was added dropwise to this solution, turning the purple mixture dark green. The solution was allowed to stir for 2 hours. Precipitates were removed by passing the solution through a 0.2 μm syringe filter and the product was precipitated from solution with dry Et_2O (50 mL) to yield a green residue. The Et_2O was decanted followed by further additions of dry Et_2O (30 mL) until a fine suspension was obtained. The suspension was collected through filtration on a sintered glass filter and subsequently dried under vacuum for 1 h. Crystals suitable for single crystal XRD were grown *via* slow layer diffusion of Et_2O into a solution of **1(OH)** in DMF. Yield: 0.65 g, 85 %. The product was stored at -35 °C under N_2, because a slow decomposition at room temperature in the solid state was noted over 3 months.

λ_{max}/nm (CH_3CN, ε/L mol^{-1} cm^{-1}): 492 (150), 614 (60), 637 (70), 785 (25).

δ_H (400 MHz, D_6-DMSO, ppm): -18.7 (s, broad, *o/m-Ar*), -6.34 (s, broad, *p-Ar*), 2.69 (s, DMF), 2.84 (s, DMF), 3.38 (s, Me_4N^+), 7.92 (*s*, DMF), 13.52 (s, broad *o/m-Ar*), 68.59 (s, broad, C*H_2*).

ESI-MS (*m*/*z*): Found: 472.0964 ([$Co^{II}(L^{Ph})]^-$, $C_{24}H_{21}CoN_4O_3$ requires 472.0946).

ν (ATR-FTIR)/cm^{-1}: 3503 (OH), 3024 (CH), 2890 (CH), 2836 (CH), 1679, 1598, 1559, 1515, 1485, 1446, 1388, 1330, 1296, 1252, 1226, 1167, 1152, 1127, 1103, 1070, 1059, 1028, 1008, 980, 954, 913, 898, 876, 823, 757, 693, 660, 645, 619, 588, 565.

Synthesis of tetramethylammonium (nitrile-tris-(N-phenylacetamide)cobalt(II)acetamide, $[NMe_4]_2[Co^{II}(L^{Ph})(NHCOCH_3)]$, ***2***:

1(DMF) (1.20 mmol) was generated as described above, but once the purple mixture formed, no further work-up was performed. A solution of $[NMe_4][OH]$ (10.4 N , 0.37 mL, 3.84 mmol, 3.2 equiv.) was added dropwise to this solution, turning the purple mixture dark green. The solution was allowed to stir for 2 h. Precipitates were removed by passing the solution through a 0.2 μm syringe filter and the product was precipitated from solution with dry Et_2O (50 mL) to yield a green residue. The pale green supernatant was decanted, leaving behind a green residue of **1(OH)**. Dry CH_3CN (10 mL) was added and the mixture was allowed to stir until most of the green product had dissolved into the solution. Any remaining precipitate was removed with the aid of a 0.2 μm syringe filter. The dark green solution was transferred into a crystalizing tube and layered with dry Et_2O. Dark purple crystals, suitable for XRD grew over a 1 week period. The crystals were filtered and washed with dry Et_2O and dried under vacuum. Yield: 0.33 g, 41 %.

λ_{max}/nm (CH_3CN, ε/L mol^{-1} cm^{-1}): 500 (160), 595 (150).

δ_H (400 MHz, D_6-DMSO, ppm): -9.18 (s, broad, *p-Ar*), -8.61 (s, broad, *o/m-Ar*), 3.08 (s, Me_4N^+), 17.39 (s, broad *o/m-Ar*), 50.61 (s, broad, C*H*$_2$).

ESI-MS (*m*/*z*): Found: 472.0961 ($[Co^{II}(L^{Ph})]^-$, $C_{24}H_{21}CoN_4O_3$ requires 472.0946).

ν (ATR-FTIR)/cm^{-1}: 3238 (NH), 3041 (CH), 2902 (CH), 2817 (CH), 1689 (CO), 1651, 1598, 1539, 1495, 1445, 1415, 1367, 1345, 1313, 1300, 1280, 1247, 1211, 1192, 1140, 1078, 1027, 986, 958, 945, 906, 856, 836, 794, 746, 715, 690, 623, 617, 606, 583, 561.

Reactivity studies:

CH_3CN solutions (5.0 mM) of **1(OAc)**, **1(OH)**, and **2** were prepared and monitored at 25 °C (Figure S10). For studying the reaction of **1(OH)** with CH_3CN, a fresh solution of **1(OH)** (5.0 mM) was prepared in CH_3CN by adding $[NMe_4][OH]$ (5.0 equiv.) to **1(OAc)** (5.0 mM, CH_3CN). To follow this reaction by electronic absorption spectroscopy, the solution was rapidly transferred it into a cuvette (2 mL) at 25 °C. The same method was followed for monitoring the reaction of **1(OH)** with benzonitrile and butyronitrile.

Catalytic studies were carried out by dissolving **1(OAc)** in CH_3CN (2 ml, 15.0 mM) under anaerobic conditions and then adding a concentrated methanolic solution of $[NMe_4][OH]$ (20.0 equiv.). The solution was stirred under nitrogen for 48 h. Subsequently, maleic acid was added as an internal standard and the solvent was removed under reduced pressure. After complete removal of CH_3CN, D_2O was added to the reaction mixture and the solution was passed through a syringe filter. The filtered solution was then analyzed by ^1H NMR to calculate the yield of the acetamide formed during reaction (Figure S18).

Table S1. Crystal data and structure refinement for **1(DMF)**, **1(OAc)**, **1(OH)**, and **2**.

Complex	**1(DMF)**	**1(OAc)**	**1(OH)**	**2**
Empirical formula	$C_{27}H_{28}CoKN_5O_4$	$C_{32}H_{46}CoN_6O_4$	$C_{36}H_{51}CoN_7O_5$	$C_{36.2}H_{51.8}CoN_8O_{4.2}$
Formula weight	584.57	637.68	720.76	725.18
Temperature (K)	105(2)	100(2)	100(2)	150(2)
Crystal system	monoclinic	monoclinic	orthorhombic	orthorhombic
Space group	$P2_1/c$	C2/c	$P2_12_12_1$	$P2_12_12_1$
a (Å)	11.1674(4)	35.9461(16)	11.1234(4)	11.2795(7)
b (Å)	11.4510(4)	11.2664(5)	17.4524(5)	17.5621(12)
c (Å)	21.0396(7)	18.3190(8)	19.3058(6)	19.5358(12)
α (°)	90	90	90	90
β (°)	98.0754(10)	99.9728(12)	90	90
γ (°)	90	90	90	90
Volume ($Å^3$)	2663.82(16)	7306.8(6)	3747.8(2)	3869.9(4)
Z	4	8	4	4
ρ_{calc} (g/cm^3)	1.458	1.159	1.277	1.245
μ (mm^{-1})	0.843	0.510	3.992	0.491
F(000)	1212.0	2712.0	1532.0	1542.0
Crystal size (mm^3)	0.196 × 0.125 × 0.055	0.405 × 0.286 × 0.28	0.242 × 0.179 × 0.12	0.314 × 0.161 × 0.113
Radiation	Mo Kα (λ = 0.71073)	Mo Kα (λ = 0.71073)	Cu Kα (λ = 1.54178)	Mo Kα (λ = 0.71073)
2Θ range for data collection (°)	6.122 to 55.152	5.744 to 55.272	6.828 to 139.956	4.17 to 54.978
Index ranges	-14 ≤ h ≤ 14, -14 ≤ k ≤ 14, -27 ≤ l ≤ 27	-46 ≤ h ≤ 46, -14 ≤ k ≤ 14, -23 ≤ l ≤ 23	-13 ≤ h ≤ 13, -20 ≤ k ≤ 21, -23 ≤ l ≤ 23	-14 ≤ h ≤ 12, -20 ≤ k ≤ 22, -25 ≤ l ≤ 25
Reflections collected	39460	55028	24599	35435
Independent reflections	6146 R_{int} = 0.0581 R_{sigma} = 0.0327	8473 R_{int} = 0.0343 R_{sigma} = 0.0207	7027 R_{int} = 0.0393 R_{sigma} = 0.0350	8879 R_{int} = 0.0709 R_{sigma} = 0.0806
Data/restraints/parameters	6146/0/351	8473/1/406	7027/0/452	8879/16/472
Goodness-of-fit on F^2	1.012	1.050	1.059	1.040
Final R* indexes [I≥2σ (I)]	R_1 = 0.0338, wR_2 = 0.0707	R_1 = 0.0356, wR_2 = 0.0847	R_1 = 0.0284, wR_2 = 0.0751	R_1 = 0.0491, wR_2 = 0.1009
Final R indexes [all data]	R_1 = 0.0536, wR_2 = 0.0790	R_1 = 0.0442, wR_2 = 0.0896	R_1 = 0.0301, wR_2 = 0.0759	R_1 = 0.0961, wR_2 = 0.1164
Largest diff. peak/hole (e $Å^{-3}$)	0.44/-0.39	1.00/-0.49	0.32/-0.26	0.77/-0.46
Flack parameter	-	-	-0.0230(14)	-0.003(8)

*$R_1 = \Sigma||F_o| - |F_c||/\Sigma|F_o|$, $wR_2 = [\Sigma w(F_o^2 - F_c^2)^2/\Sigma w(F_o^2)^2]^{1/2}$.

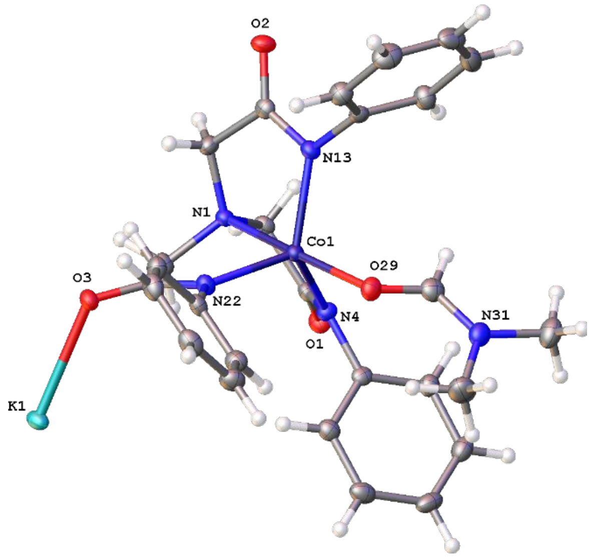

Figure S1. 1(DMF) with displacement parameters shown at 50% probability with heteroatoms labelled only.

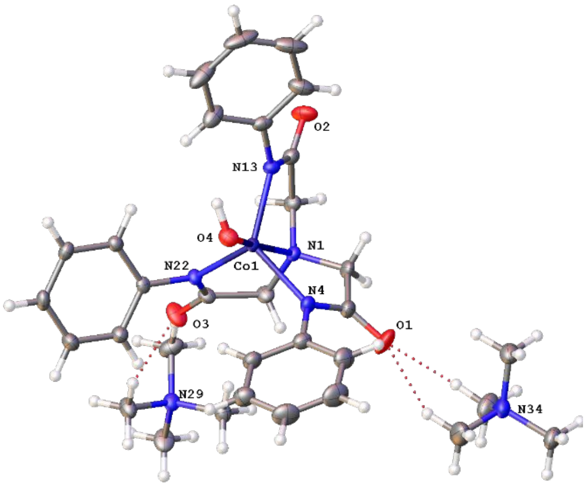

Figure S2. 1(OH) with displacement parameters shown at 50% probability with heteroatoms labelled only.

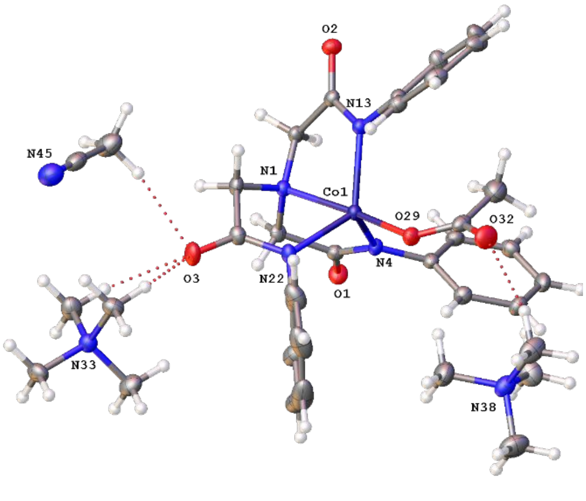

Figure S3. 1(OAc) with displacement parameters shown at 50% probability with heteroatoms labelled only.

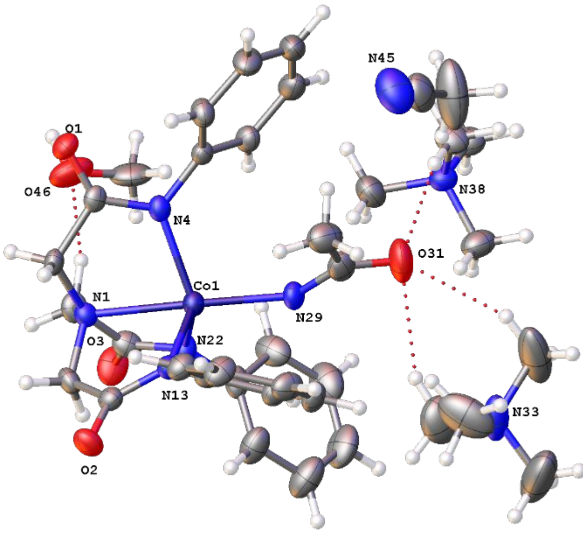

Figure S4. 2 with displacement parameters shown at 50% probability with heteroatoms labelled only.

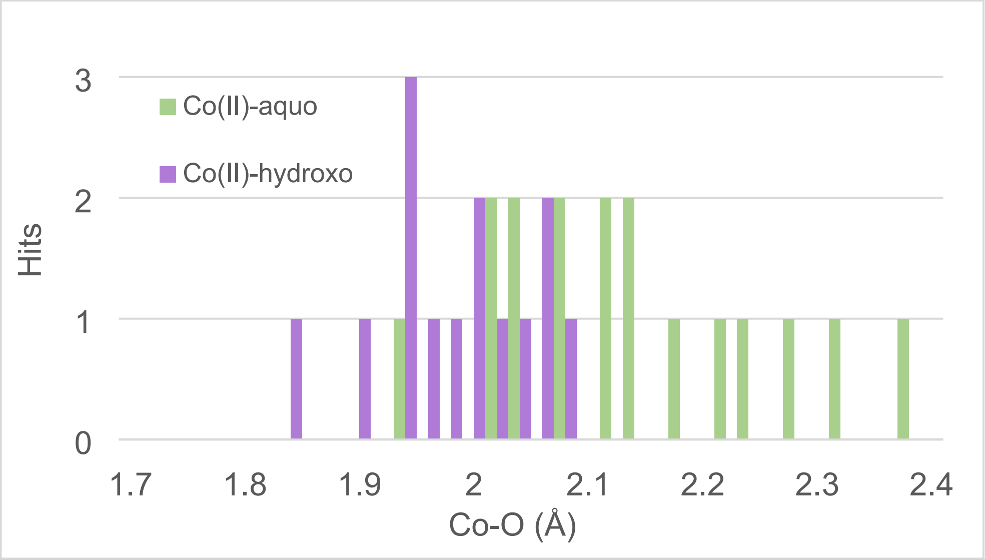

Figure S5. CCDC Library search for Co^{II}–OH (purple) and –OH_2 (green) complexes supported by tetradentate N-donor ligands.[9]

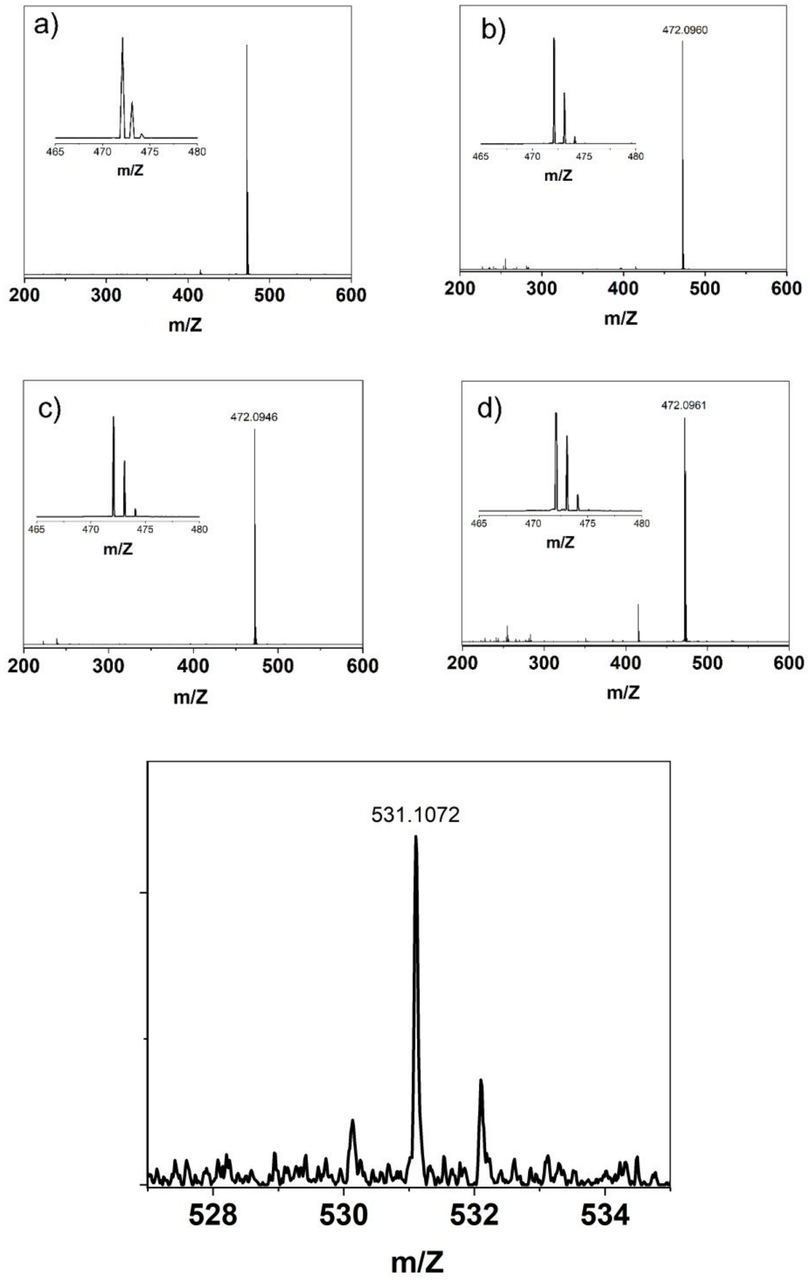

Figure S6. Negative mode ESI-MS of **1(DMF)** (a), **1(OAc)** (b), **1(OH)** (c, and 5th image showing the *m/Z* = 531.1079 ion assigned to [Co^{III}(L^{Ph})(OAc)]$^{-}$) and **2** (d).

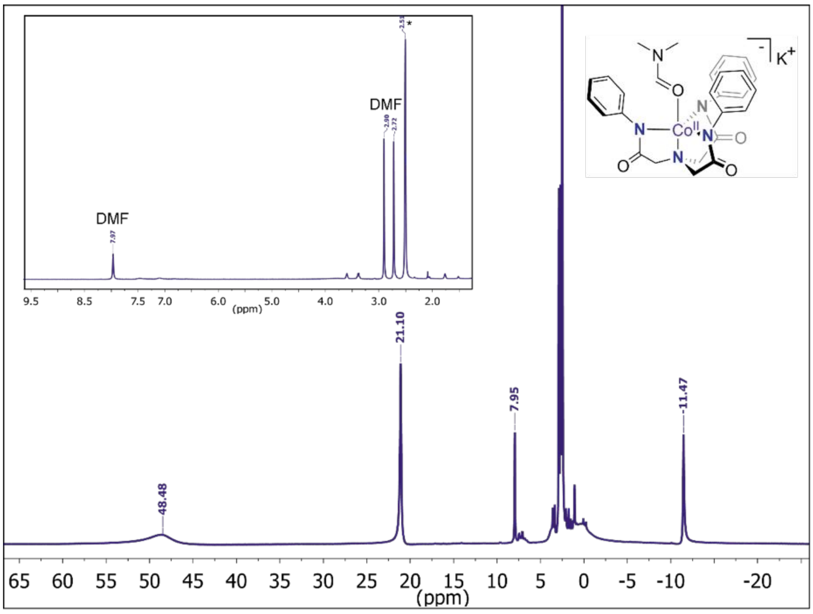

Figure S7. ^{1}H NMR spectrum of **1(DMF)** in $[D]_6$-DMSO at 20 °C. *Residual solvent peak.

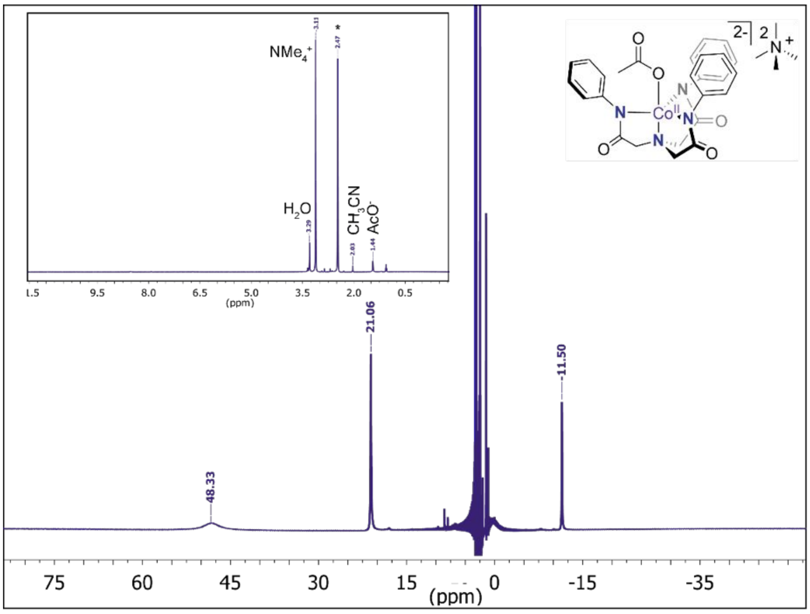

Figure S8. ^{1}H NMR spectrum of **1(OAc)** in $[D]_6$-DMSO at 20 °C. *Residual solvent peak.

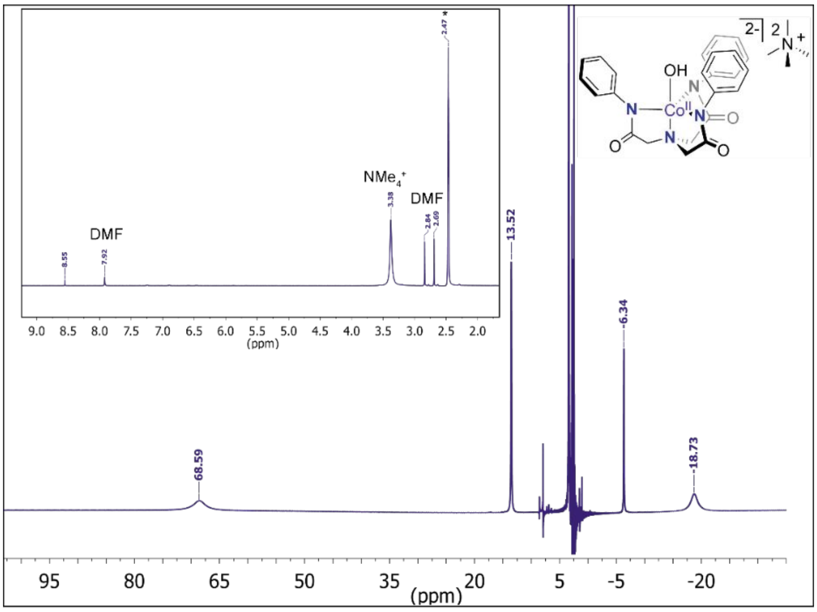

Figure S9. ^{1}H NMR spectrum of **1(OH)** in $[D]_6$-DMSO at 20 °C. *Residual solvent peak.

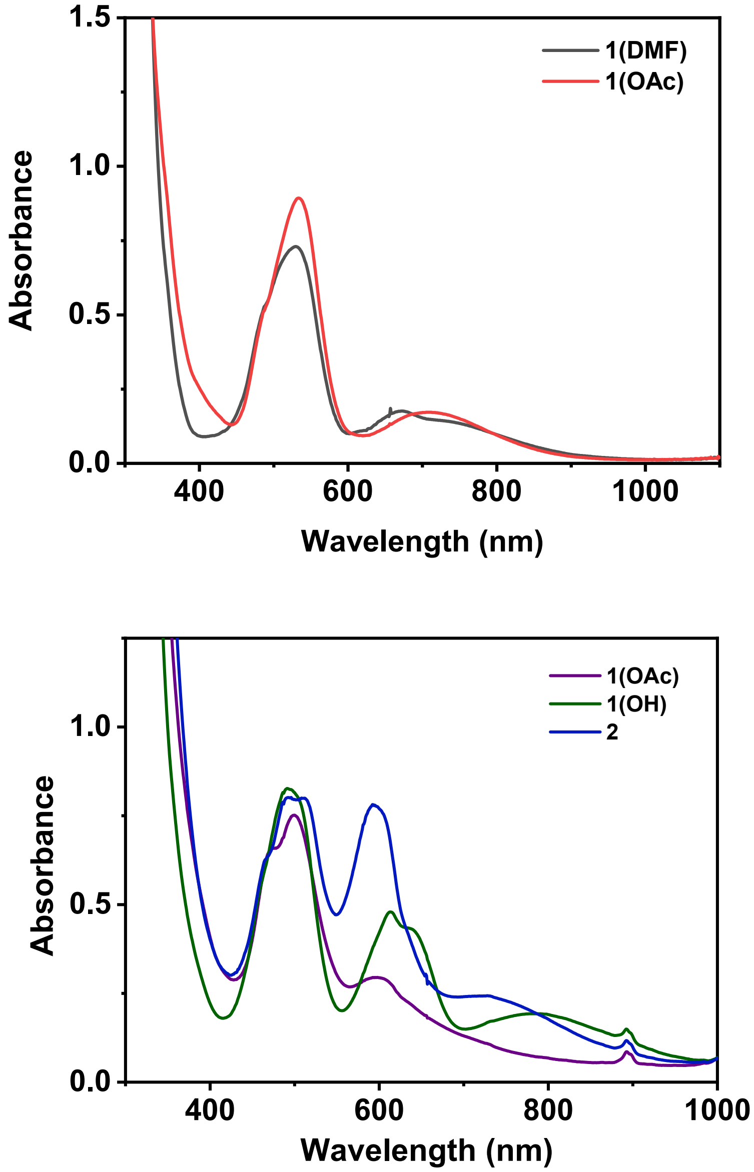

Figure S10. ***Top:*** Electronic absorption spectra of **1(DMF)** and **1(OAc)** (red trace). Measured at 20 °C in DMF, both at 5.0 mM. ***Bottom:*** Electronic absorption spectra of **1(OAc)** (purple trace), **1(OH)** (green trace) and **2** (blue trace). Measured at 20 °C in CH_3CN, all at 5.0 mM. **1(DMF)** not shown because insoluble in CH_3CN.

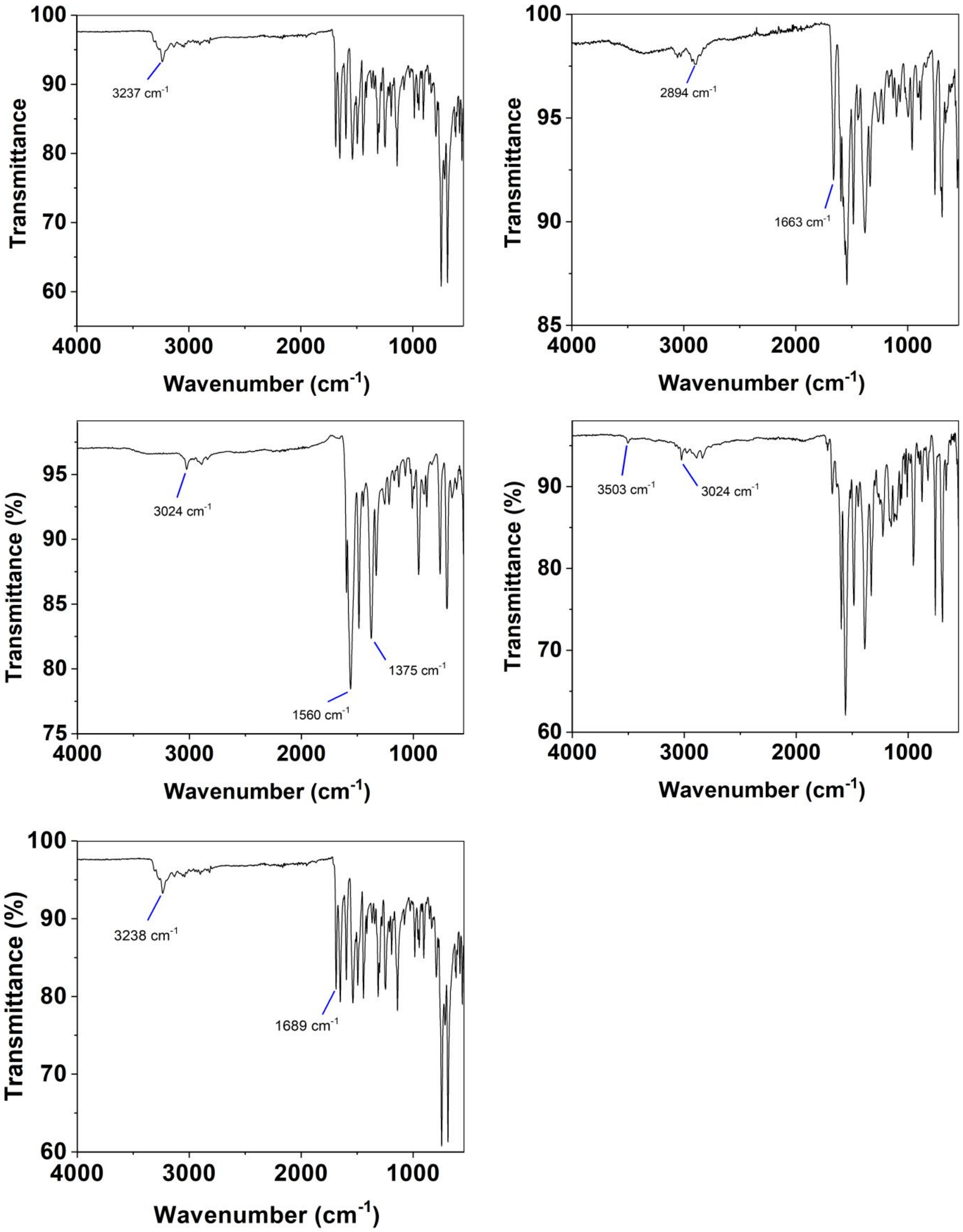

Figure S11. ATR-FTIR spectra of powdered samples of L^{Ph} (top left), **1(DMF)** (top right), **1(OAc)** (middle left), **1(OH)** (middle right) and **2** (bottom left). Measured at 20 °C.

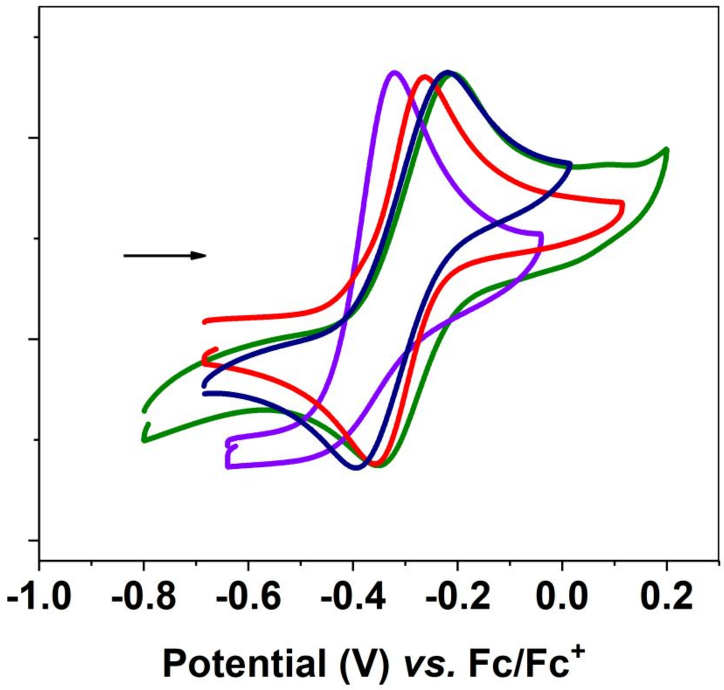

Figure S12. Steady state cyclic voltammogram of **1(DMF)** (red trace), **1(OAc)** (purple trace) **1(OH)** (green trace) and **2** (blue trace) measured at room temperature in DMF using 1.0 mM concentrations. Measured at 20 °C. Scan rate: 0.05 V s^{-1}, 0.1 N [$^{n}Bu_4N$][PF_6] as supporting electrolyte. Arrow indicates sweep direction.

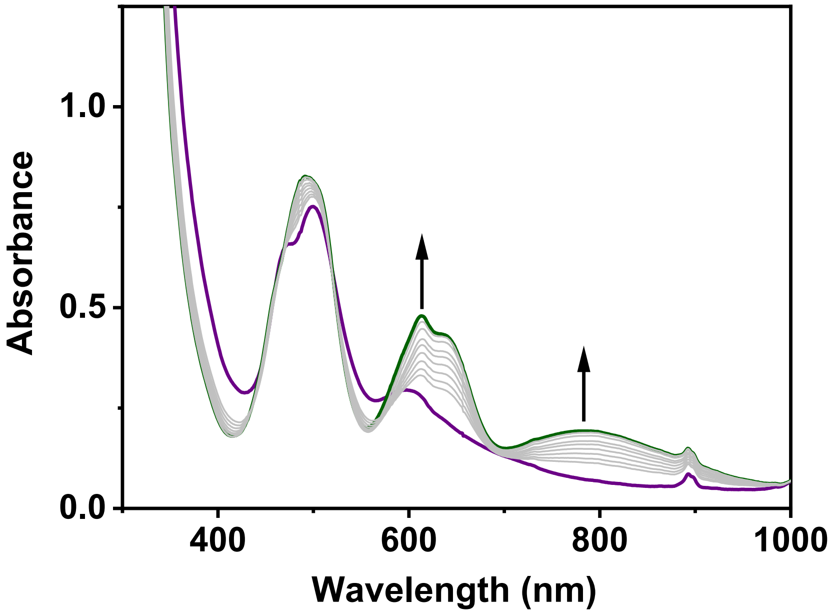

Figure S13. Electronic absorption spectra of the generation of **1(OH)** (green trace) upon addition of OH^- (5 equiv.) to a CH_3CN solution of **1(OAc)** (purple trace). Measured at 5.0 mM of **1(OAc)** in CH_3CN at 20 °C.

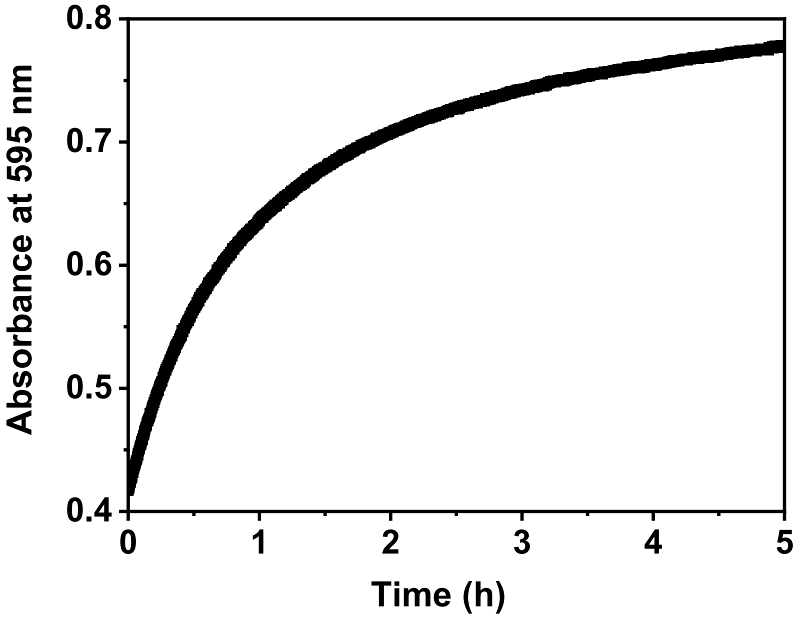

Figure S14. Time trace monitoring changes in the electronic absorption spectrum at λ = 595 nm for the conversion of **1(OH)** (5.0 mM) into **2** in CH_3CN at 20 °C.

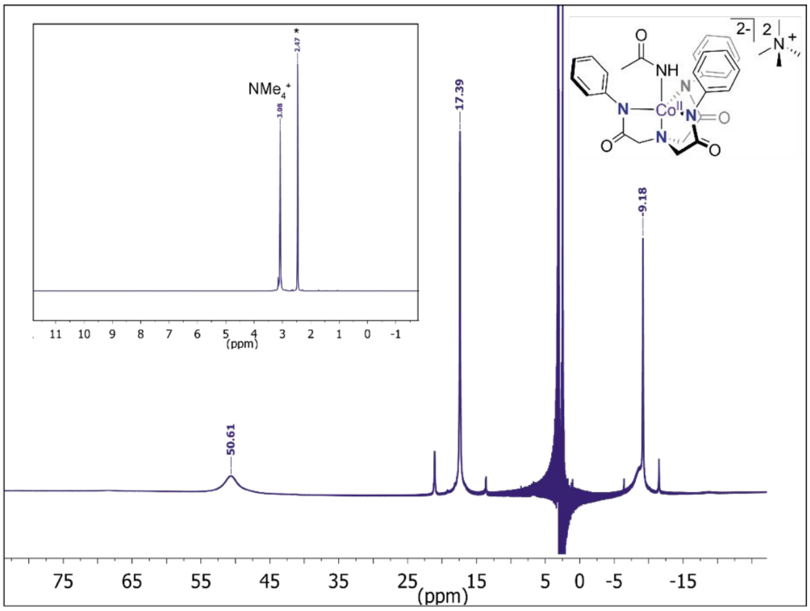

Figure S15. ^{1}H NMR spectrum of **2** in [D]$_6$-DMSO at 20 °C. *Residual solvent peak.

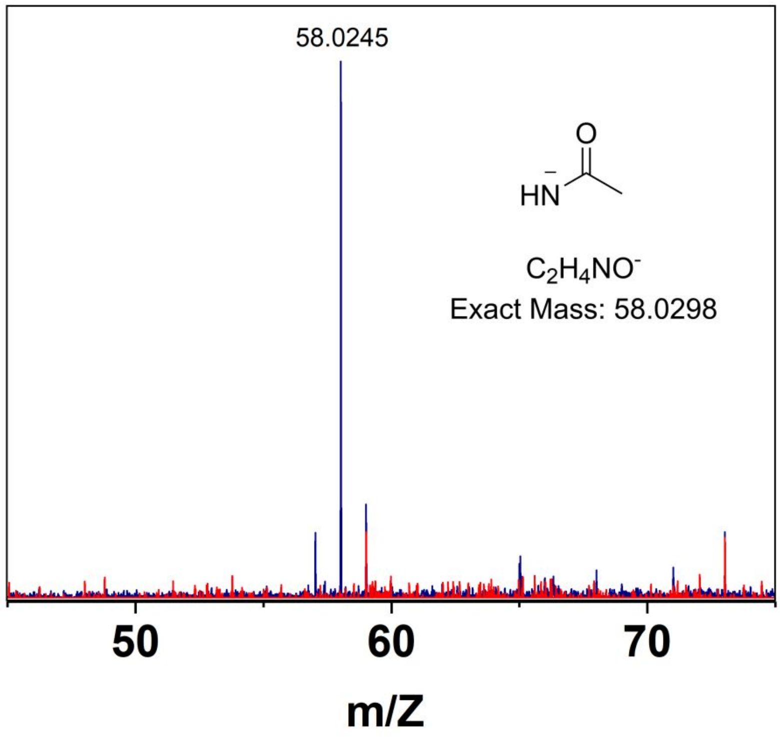

Figure S16. Negative mode ESI-MS of **2** (blue trace) and **1(OAc)** (red trace). The peak at m/Z = 58 can be assigned to free acetamide, not observed in the ESI-MS of **1(OAc)**.

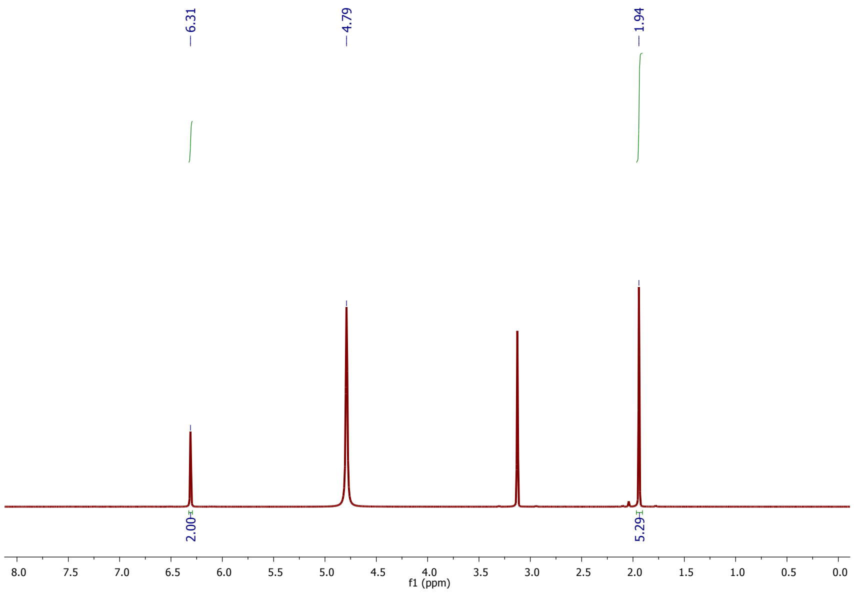

Figure S17. ^{1}H NMR spectrum of the post-reaction mixture in D_2O obtained after the reaction of **1(OH)** (0.025 mmol) with $[NMe_4][OH]$ (0.5 mmol) in acetonitrile (solvent) after 24 h of stirring at 20 °C. The signal at δ = 6.3 ppm corresponds to the internal standard maleic acid (0.3 mmol) and the signal at δ = 1.9 ppm corresponds to the released free acetamide.

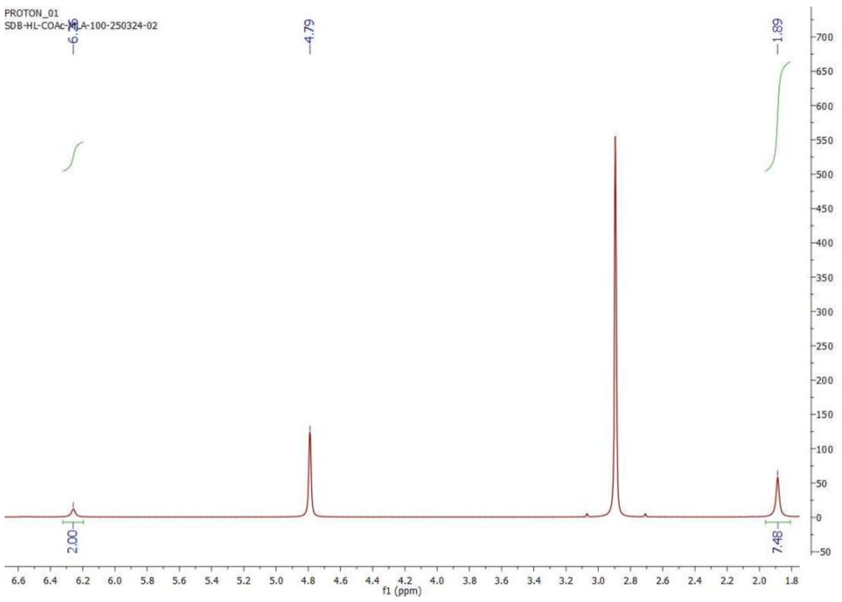

Figure S18. ^{1}H NMR spectrum of the post-reaction mixture in D_2O obtained after the reaction of **1(OH)** (0.025 mmol) with $[NMe_4][OH]$ (2.5 mmol) in acetonitrile (solvent) after 65 h of stirring at 20 °C. The signal at δ = 6.3 ppm corresponds to the internal standard maleic acid (1 mmol) and the signal at δ = 1.9 ppm corresponds to the released free acetamide.

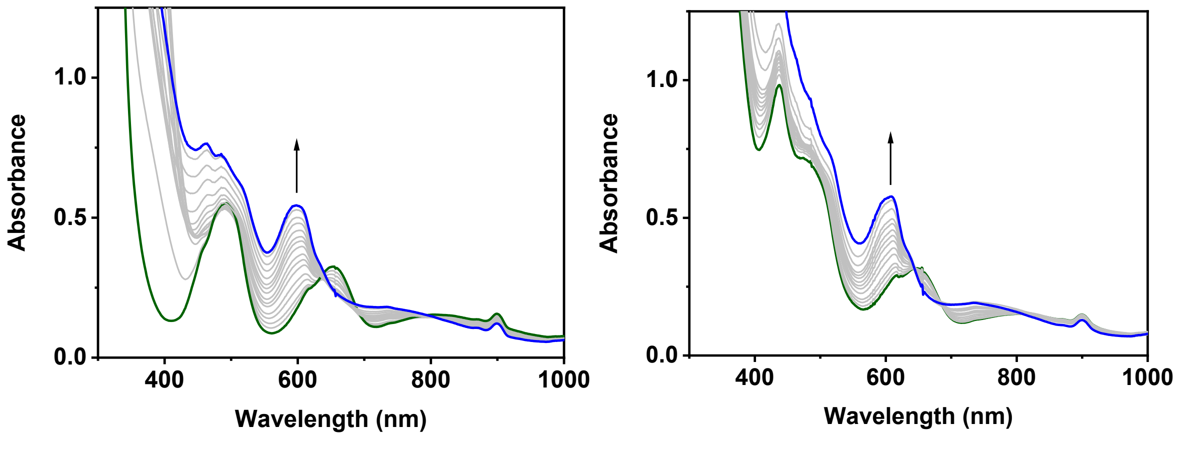

Figure S19. Electronic absorption spectral changes of the reaction between **1(OH)** (green trace, 5.0 mM) with benzonitrile (left) and butyronitrile (right) at 20 °C in benzonitrile and butyronitrile as solvent, respectively. Grey traces show incremental changes in the electronic

absorption, blue traces show the end of the reaction, comparable to that obtained for **2**, indicating nitrile hydration.

References

(1) Loliger, J.; Scheffold, R. Paramagnetic moment measurements by NMR. A micro technique. *J. Chem. Educ.* **1972**, *49* (9), 646.
(2) Bruker. APEX3 v2017.3-0. *, Bruker AXS Inc., Madison, WI, USA.* **2017**.
(3) Krause, L.; Herbst-Irmer, R.; Sheldrick, G. M.; Stalke, D. Comparison of silver and molybdenum microfocus X-ray sources for single-crystal structure determination. *J. Appl. Cryst.* **2015**, *48* (1), 3-10.
(4) Sheldrick, G. Crystal structure refinement with SHELXL. *Acta Cryst. A* **2015**, *71* (1), 3-8.
(5) Sheldrick, G. Crystal structure refinement with SHELXL. *Acta Cryst. C* **2015**, *71* (1), 3-8.
(6) Dolomanov, O. V.; Bourhis, L. J.; Gildea, R. J.; Howard, J. A. K.; Puschmann, H. OLEX2: a complete structure solution, refinement and analysis program. *J. Appl. Cryst.* **2009**, *42* (2), 339-341.
(7) Spek, A. PLATON SQUEEZE: a tool for the calculation of the disordered solvent contribution to the calculated structure factors. *Acta Cryst. C* **2015**, *71* (1), 9-18.
(8) Rowland, J. M.; Ibanez, G. J.; Olmstead, M. M.; Ruf, M.; Mascharak, P. K. Nitrilotriacetanilide. *Acta Cryst. E* **2001**, *57* (11), o1001-o1003.
(9) Groom, C. R.; Bruno, I. J.; Lightfoot, M. P.; Ward, S. C. The Cambridge structural database. *Acta Cryst. B* **2016**, *72* (2), 171-179.
